# Clinico–pathologic factors and survival of patients with breast cancer diagnosed with de novo brain metastasis: a national cancer database analysis

**DOI:** 10.1007/s10549-024-07321-x

**Published:** 2024-04-29

**Authors:** Ali Hijazi, Mohamed Mohanna, Saad Sabbagh, María Herrán, Barbara Dominguez, Kaylee Sarna, Zeina Nahleh

**Affiliations:** 1 Department of Hematology and Oncology, Cleveland Clinic Florida, 2950 Cleveland Clinic Blvd, Weston, FL 33331 USA; 2 Center for Clinical Research, Cleveland Clinic Foundation, Weston, FL 33331 USA

**Keywords:** Breast cancer, Brain metastasis, Immunotherapy, Survival, Prognosis, NCDB

## Abstract

**Purpose:**

Patients with Breast Cancer (BC) with Brain Metastasis (BCBM) have poor survival outcomes. We aimed to explore the clinico–pathologic and therapeutic factors predicting the survival in patients with de novo BCBM using the National Cancer Database (NCDB).

**Patients and methods:**

The NCDB was queried for patients with BC between 2010 and 2020. Survival analysis with Kaplan–Meier curves and log rank tests were used to find median overall survival (OS) in months (95% CI) across the different variables. A multivariate cox regression model was computed to identify significant predictors of survival.

**Results:**

Out of n = 2,610,598 patients, n = 9005 (0.34%) had de novo BCBM. A trend of decreasing OS was observed with increasing age, Charlson–Deyo score (CDS), and number of extracranial metastatic sites. The highest median OS was observed in the Triple Positive and the lowest OS in the Triple Negative subgroup. Based on treatment regimen, combination of systemic therapy and local therapy achieved the highest OS. A positive trend in OS was observed in the BC subgroup analysis with targeted therapy demonstrating a survival benefit when added to systemic therapy.

The multivariate cox regression model showed that age, race, ethnicity, insurance, median income, facility type, CDS, BC subtype, metastatic location sites, and treatment combinations received were significantly associated with risk of death. Receiving only local treatment for BM without systemic therapy more than doubled the risk of death compared to combining it with systemic therapy.

**Conclusions:**

This analysis suggests that treatment of systemic disease is the major factor influencing survival in patients with BCBM. Moreover, targeted therapy with anti–HER2 increased survival when added to systemic therapy explaining the highest median OS noted in the Triple Positive subgroup.

**Supplementary Information:**

The online version contains supplementary material available at 10.1007/s10549-024-07321-x.

## Introduction

**Fig. 1:**
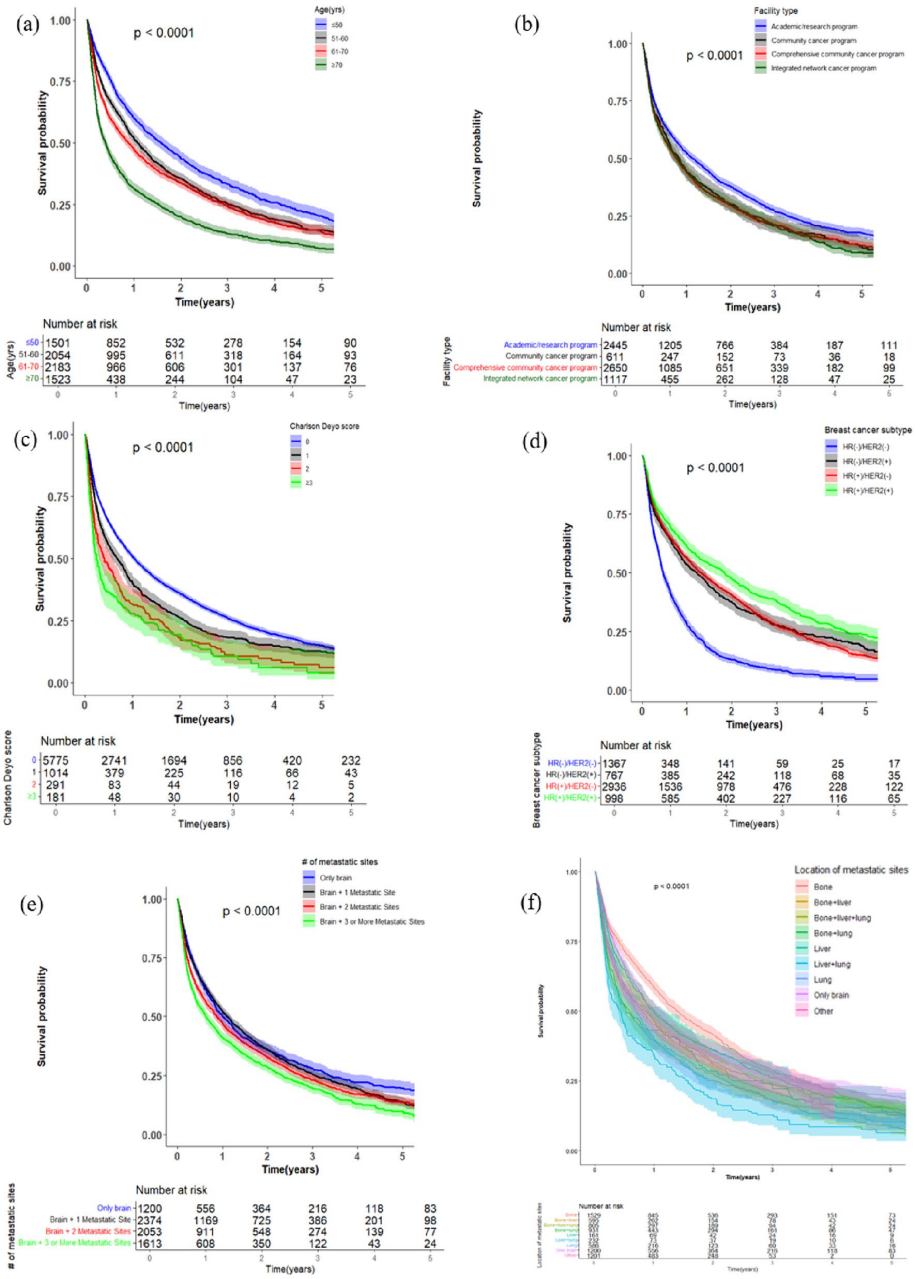
Kaplan–Meier plots of overall survival for breast cancer patients with brain metastases stratified by **a** age, **b** facility type, **c** Charlson-Deyo score, **d** breast cancer subtype, **e** number of extracranial metastatic sites, and **f** location of extracranial metastatic sites

**Fig. 2:**
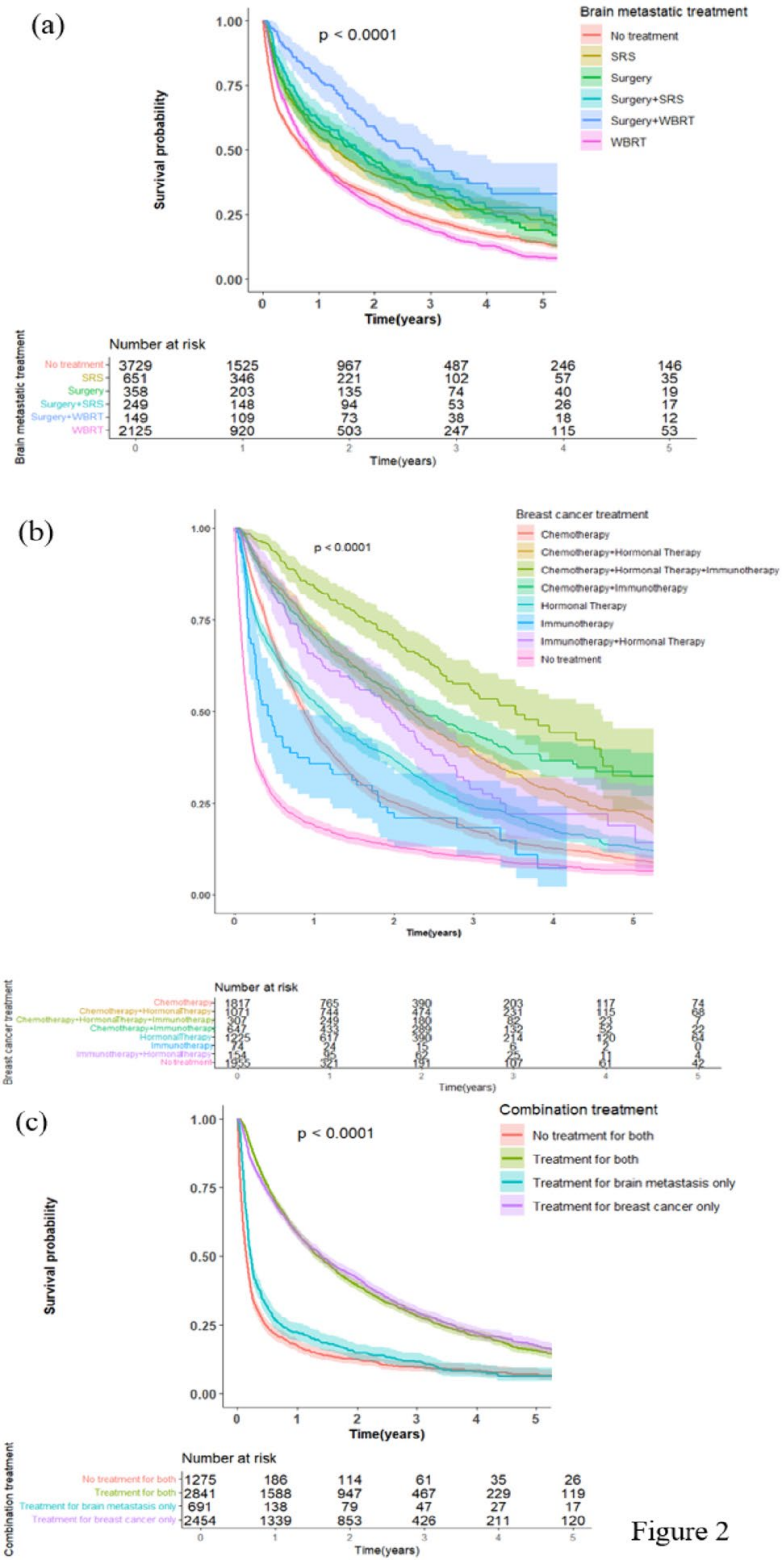
Kaplan–Meier plots of overall survival for breast cancer patients with brain metastases stratified by **a** brain metastases treatment modality, **b** breast cancer treatment modality, and **c** combination of both breast cancer and brain metastases treatments

Breast Cancer (BC) ranks as the most common malignancy among females worldwide with an annual incidence of 2.3 million cases [1, 2]. Specifically, BC with metastasis at diagnosis (de novo metastatic BC) comprises 3–6% of all BC patients and presents a major clinical challenge as these patients have limited–life expectancy [3], with an estimated five–year survival of metastatic BC in women residing in the US limited to 30% [4]. The most common sites of BC metastases include bone, liver, lung, and brain, of which the brain metastatic group has the worst survival outcomes [5]. BC is the second most common source of brain metastases (BM) after lung cancer [6]. The incidence of breast cancer brain metastases (BCBM) has increased steadily over the last several years owing to improved management of the primary disease [7]. Many studies have explored the factors that might predict survival in patients with BCBM, with many factors identified including age, race, marital status, histology, grade, tumor size, molecular subtype, patterns of metastasis, history of chemotherapy, radiotherapy, and surgery of primary cancer [8, 9]. Such studies have led to the development of prognostic scores that help in clinical decision making, such as the well–studied Graded Prognostic Assessment (GPA) scoring tool, which was developed to estimate survival in different BM patients based on the tumor of origin [10]. Some of the significant factors used in the score include age, Karnofsky Performance Status (KPS), extracranial metastases, and number of BM [2]. According to the National Comprehensive Cancer Network (NCCN) guidelines, treatment of BM includes surgery for relief of symptoms, whole brain radiotherapy (WBRT), stereotactic radiosurgery (SRS), and palliative care if applicable [11]. Additionally, BCBM require treatment based on the primary tumor characteristics including chemotherapy, hormonal, and anti–HER2 targeted therapy [12, 13]. There is a growing number of studies and clinical trials investigating newer targeted therapies for BCBM which span different classes such as EGFR receptor modulators, tyrosine kinase inhibitors, and CDK4/6 inhibitors to name a few [14–22]. Despite our growing knowledge about BCBM and the many efforts to identify prognostic and therapeutic interventions, large population–based survival studies on de novo BCBM remain lacking. Therefore, we aim to retrospectively analyze the national cancer database (NCDB) to identify factors and therapeutic interventions predicting survival of patients presenting with BCBM (Figs. 1 and 2).

## Materials and methods

### Patient data

The NCDB was queried for patients with BC with available data on de novo BM between 2010 and 2020. A total of n = 2,610,598 records of patients with BC were identified, out of whom 9005 had de novo BM. Access to this registry was achieved based on a Participant User File award granted to the principal investigator (N.Z.). The NCDB is a clinical oncology database sourced from hospital registry data collected in more than 1500 Commission on Cancer–accredited facilities (amounting to about 70% of all cancer diagnoses in the country). These data are used to analyze and track patients with malignant neoplastic diseases, their treatments, and outcomes. Variables used from the dataset included facility and patient demographics, BC–specific variables, and treatment modalities. Several variables were computed that are relevant to prognosis in this patient population. Variables for the number and location of extracranial metastatic sites (EMS) were computed by combining five individual metastatic sites: bone, liver, lung, distant lymph nodes, and other sites. A variable on BM treatment modality was computed by combining three individual modalities: Surgery, WBRT, and SRS. A variable on BC treatment modality was computed by combing three individual modalities: chemotherapy, hormonal therapy, and immunotherapy (referring to anti–HER2 therapy and other targeted therapies). Last, a variable on treatment combination was computed by combining the treatment status for BM and BC.

### Statistical analysis

Chi–square, fisher exact, independent t, and Mann Whitney U tests were performed to evaluate the association between each categorical variable and treatment combinations received. Kaplan–Meier analyses and log rank tests were performed on the whole dataset to compare median overall survival (OS) across age, facility type, Charlson–Deyo Score (CDS), BC subtype, number of EMS, location of EMS, BM treatment modality, BC treatment modality, and combination of both treatment modalities. Furthermore, the same analysis was conducted on the four BC subgroups to compare OS across the different treatment modalities. Finally Univariate and Multivariate Cox regression models were computed with backwards elimination of 0.1 for both to identify the significant predictors of survival in the patient cohort. The cutoff of statistical significance was set at p < 0.05. SAS version 9.4 and R 4.2.3 were used for data analysis.

## Results

### Baseline characteristics

Out of n = 2,610,598 patients identified with BC in the NCDB between 2010 and 2020, n = 9005 (0.34%) patients had de novo BM. Table 1 outlines the baseline characteristics of this cohort across the different treatment combinations received. Most patients with de novo BM were in the 61–70 age (30.3%) compared to the lowest proportion in the ≤ 50 age group (20.6%). Most patients were female (98.9%), of White race (76.6%), and non–Hispanic ethnicity (92.4%). Most patients were treated at either Comprehensive Community Cancer Programs (CCCP) (39.2%) or Academic/Research Programs (35.5%). In this database, most patients had insurance with only 7.5% of the cohort being un–insured. Most patients had a CDS of 0 (79.7%) with only 2.6% having a score of ≥ 3. There was a trend of increasing BCBM diagnosis during the 11–year span ranging from 7.7% in 2010 to 10.2% in 2020. Most BC cases had invasive ductal histology (64.9%), were poorly differentiated (43.4%), and ≥ 3 cm in size (62.8%). The BC subtype proportions in this cohort were as follows: 48% HR( +)/HER2( − ), 22.6% HR( − )/HER2( − ), 16.8% HR( +)/HER2( +), and 12.6% HR(−)/HER2( +). Based on the number of EMS, 15.7%, 31.3%, 27.9%, and 25.1% of the cohort had 0, 1, 2, and ≥ 3 EMS, respectively. 17.7% of the patients did not receive treatment for either BC or BM, 9.5% received treatment for BM only, 33.6% received treatment for BC only, and 39.1% received treatment for both BC and BM. All variables except sex, ethnicity, facility type, year of diagnosis, and lympho–vascular invasion were significantly associated with the treatment combination received (p < 0.005).

**Table 1:** Baseline demographics and breast cancer-related variables with group comparisons across the different treatments received

Variable	Categories	Overall	No treatment for both	Treatment for BM only	Treatment for BC only	Treatment for both	p-value
N (%)		9005 (100)	1594 (17.7%)	859 (9.5%)	3027 (33.6%)	3525 (39.1%)	
Age (years) N (%)	≤ 50	1854 (20.6)	187 (11.7)	123 (14.3)	691 (22.8)	853 (24.2)	< 0.001
	51–60	2510 (27.9)	341 (21.4)	232 (27.0)	873 (28.8)	1064 (30.2)	
	61–70	2732 (30.3)	470 (29.5)	276 (32.1)	912 (30.1)	1074 (30.5)	
	≥ 70	1909 (21.2)	596 (37.4)	228 (26.5)	551 (18.2)	534 (15.1)	
Sex N (%)	Female	8904 (98.9)	1577 (98.9)	852 (99.2)	2993 (98.9)	3482 (98.8)	0.7821
	Male	101 (1.1)	17 (1.1)	7 (0.8)	34 (1.1)	43 (1.2)	
Race N (%), n = 8919	Black	1671 (18.7)	329 (20.9)	187 (22.1)	522 (17.4)	633 (18.1)	0.0071
	Other	415 (4.7)	69 (4.4)	32 (3.8)	154 (5.1)	160 (4.6)	
	White	6833 (76.6)	1177 (74.7)	628 (74.1)	2329 (77.5)	2699 (77.3)	
Ethnicity N (%), n = 8764	Hispanic	664 (7.6)	106 (6.9)	61 (7.3)	241 (8.2)	256 (7.5)	0.4304
	Non-Hispanic	8100 (92.4)	1434 (93.1)	778 (92.7)	2707 (91.8)	3181 (92.5)	
Insurance status N (%), n = 8768	Medicaid	1439 (16.4)	206 (13.4)	114 (13.7)	512 (17.4)	607 (17.6)	< 0.0001
	Medicare	3332 (38.0)	800 (51.9)	400 (48.0)	1025 (34.9)	1107 (32.0)	
	Not insured	655 (7.5)	144 (9.3)	67 (8.0)	215 (7.3)	229 (6.6)	
	Private insurance/managed care	3342 (38.1)	391 (25.4)	253 (30.3)	1183 (40.3)	1515 (43.8)	
Facility type N (%), n = 8449	Academic/research program	3003 (35.5)	495 (32.1)	300 (35.8)	998 (35.8)	1210 (36.8)	0.0976
	Community cancer program	742 (8.8)	149 (9.7)	79 (9.4)	253 (9.1)	261 (8.0)	
	Comprehensive community cancer program	3315 (39.2)	643 (41.7)	323 (38.6)	1085 (38.9)	1264 (38.5)	
	Integrated network cancer program	1389 (16.4)	254 (16.5)	135 (16.1)	451 (16.2)	549 (16.7)	
Median income quartiles 2012–2016 N (%), n = 8092	< $40,227	1758 (21.7)	337 (23.3)	179 (23.2)	581 (21.2)	661 (21.0)	0.0096
	$4022–$50,353	1810 (22.4)	327 (22.6)	186 (24.1)	580 (21.3)	717 (22.8)	
	$50,354–$63,332	1901 (23.5)	354 (24.5)	168 (21.8)	609 (22.3)	770 (24.5)	
	> $63,333	2623 (32.4)	428 (29.6)	238 (30.9)	960 (35.2)	997 (31.7)	
Percent no high school degree quartiles 2012–2016 N (%), n = 8112	< 6.3%	1675 (20.6)	265 (18.3)	138 (17.8)	602 (22.0)	670 (21.2)	0.0025
	6.3%–10.8%	2204 (27.2)	382 (26.4)	188 (24.3)	751 (27.5)	883 (28.0)	
	10.9%–17.5%	2189 (27.0)	402 (27.8)	224 (28.9)	730 (26.7)	833 (26.4)	
	> 17.6%	2044 (25.2)	398 (27.5)	224 (28.9)	651 (23.8)	771 (24.4)	
Year of diagnosis N (%)	2010	696(7.7)	127(8.0)	72(8.4)	240(7.9)	257(7.3)	0.1797
	2011	741(8.2)	135(8.5)	80(9.3)	238(7.9)	288(8.2)	
	2012	728(8.1)	132(8.3)	73(8.5)	261(8.6)	262(7.4)	
	2013	756(8.4)	117(7.3)	74(8.6)	271(9.0)	294(8.3)	
	2014	806(9.0)	123(7.7)	71(8.3)	293(9.7)	319(9.1)	
	2015	851(9.5)	150(9.4)	77(9.0)	308(10.2)	316(9.0)	
	2016	845(9.4)	163(10.2)	60(7.0)	269(8.9)	353(10.0)	
	2017	824(9.2)	156(9.8)	71(8.3)	282(9.3)	315(8.9)	
	2018	916(10.1)	171(10.7)	88(10.2)	289(9.5)	368(10.4)	
	2019	923(10.2)	157(9.9)	96(11.2)	285(9.4)	385(10.9)	
	2020	919(10.2)	163(10.2)	97(11.2)	291(9.6)	368(10.4)	
Histology N (%)	Ductal	5844 (64.9)	851 (53.4)	518 (60.3)	2044 (67.5)	2431 (69.0)	< 0.001
	Lobular	590 (6.6)	90 (5.6)	39 (4.5)	241 (8.0)	220 (6.2)	
	Other	2571 (28.6)	653 (41.0)	302 (35.2)	742 (24.5)	874 (24.8)	
Grade N (%), n = 6085	1	916(15.1)	131(14.8)	61(11.8)	383(17.4)	341(13.7)	< 0.001
	2	2525(41.5)	348(39.4)	186(36.1)	924(42.0)	1067(43.0)	
	3	2644(43.4)	405(45.8)	269(52.1)	894(40.6)	1076(43.3)	
Tumor size N (%), n = 6561	< 1 cm	538 (8.2)	74 (7.2)	53 (8.8)	175 (7.8)	236 (8.8)	0.0002
	1–2 cm	909 (13.9)	145 (14.0)	105 (17.4)	279 (12.3)	380 (14.2)	
	2–3 cm	989 (15.1)	148 (14.3)	116 (19.3)	360 (16.0)	365 (13.7)	
	> 3 cm	4125 (62.8)	666 (64.5)	328 (54.5)	1443 (63.9)	1688 (63.2)	
Lympho-vascular invasion N (%), n = 2711	0	1742(64.3)	237(63.5)	134(61.2)	655(66.0)	716(63.5)	0.4583
	1	969(35.7)	136(36.5)	85(38.8)	337(34.0)	411(36.5)	
Charlson Deyo score N (%)	0	7178 (79.7)	1196 (75.0)	637 (74.2)	2477 (81.8)	2868 (81.4)	< 0.001
	1	1222 (13.6)	232 (14.6)	138 (16.1)	401 (13.2)	451 (12.8)	
	2	370 (4.1)	91 (5.7)	54 (6.3)	90 (3.0)	135 (3.8)	
	≥ 3	235 (2.6)	75 (4.7)	30 (3.5)	59 (1.9)	71 (2.0)	
Breast cancer subtype N (%), n = 7563	HR ( − )/HER2 ( − )	1708 (22.6)	319 (30.0)	285 (42.3)	422 (15.7)	682 (21.7)	< 0.001
	HR ( − )/HER2 ( +)	956 (12.6)	122 (11.5)	91 (13.5)	285 (10.6)	458 (14.6)	
	HR ( +)/HER2 ( − )	3627 (48.0)	482 (45.3)	208 (30.9)	1514 (56.4)	1423 (45.3)	
	HR ( +)/HER2 ( +)	1272 (16.8)	140 (13.2)	90 (13.3)	464 (17.3)	578 (18.4)	
Number of extracranial metastatic sites N (%), n = 8979	Brain + 1 metastatic site	2808 (31.3)	476 (30.1)	241 (28.3)	1037 (34.3)	1054 (29.9)	< 0.001
	Brain + 2 metastatic sites	2504 (27.9)	397 (25.1)	211 (24.8)	947 (31.3)	949 (27.0)	
	Brain + ≥ 3 metastatic sites	2258 (25.1)	450 (28.5)	175 (20.5)	776 (25.7)	857 (24.3)	
	Only brain	1409 (15.7)	258 (16.3)	225 (26.4)	265 (8.7)	661 (18.8)	
Location of extracranial metastatic sites N (%), n = 8979	Bone	1786 (19.8)	283 (17.9)	109 (12.8)	799 (26.4)	595 (16.9)	< 0.001
	Bone + liver	696 (7.8)	102 (6.4)	53 (6.2)	314 (10.4)	227 (6.5)	
	Bone + liver + lung	908 (10.1)	183 (11.6)	63 (7.4)	331 (10.9)	331 (9.4)	
	Bone + lung	1038 (11.6)	169 (10.7)	71 (8.3)	380 (12.6)	418 (11.9)	
	Liver	184 (2.1)	30 (1.9)	18 (2.1)	61 (2.0)	75 (2.1)	
	Liver + lung	264 (2.9)	50 (3.2)	36 (4.2)	75 (2.5)	103 (2.9)	
	Lung	689 (7.7)	141 (8.9)	91 (10.7)	137 (4.5)	320 (9.1)	
	Only brain	1409 (15.7)	258 (16.3)	225 (26.4)	265 (8.8)	661 (18.8)	
	Other	2005 (22.3)	365 (23.1)	186 (21.8)	663 (21.9)	791 (22.4)	
Brain metastasis treatment modality N (%), n = 9004	No treatment	4620 (51.3)	1594 (100.0)	0 (0.0)	3026 (100.0)	0 (0.0)	< 0.001
	SRS	827 (9.2)	0 (0.0)	122 (14.2)	0 (0.0)	705 (20.0)	
	WBRT	2596 (28.8)	0 (0.0)	505 (58.7)	0 (0.0)	2091 (59.3)	
	Surgery	445 (4.9)	0 (0.0)	150 (17.5)	0 (0.0)	295 (8.4)	
	Surgery + SRS	311 (3.5)	0 (0.0)	53 (6.2)	0 (0.0)	258 (7.3)	
	Surgery + WBRT	205 (2.3)	0 (0.0)	29 (3.4)	0 (0.0)	176 (5.0)	
Breast cancer treatment modality N (%), n = 8991	No treatment	2439 (27.1)	1586 (100.0)	853 (100.0)	0 (0.0)	0 (0.0)	< 0.001
	Immunotherapy	98 (1.1)	0 (0.0)	0 (0.0)	49 (1.6)	49 (1.4)	
	Chemotherapy	2101 (23.4)	0 (0.0)	0 (0.0)	894 (29.5)	1207 (34.2)	
	Hormonal therapy	1434 (16.0)	0 (0.0)	0 (0.0)	753 (24.9)	681 (19.3)	
	Immunotherapy + hormonal therapy	199 (2.2)	0 (0.0)	0 (0.0)	112 (3.7)	87 (2.5)	
	Chemotherapy + hormonal therapy	1390 (15.5)	0 (0.0)	0 (0.0)	695 (23.0)	695 (19.7)	
	Chemotherapy + immunotherapy	912 (10.1)	0 (0.0)	0 (0.0)	340 (11.2)	572 (16.2)	
	Chemotherapy + hormonal therapy + immunotherapy	418 (4.6)	0 (0.0)	0 (0.0)	184 (6.1)	234 (6.6)	

### Median OS of the Cohort across different variables

The median OS of the 9005–patient cohort was 10.9 months (95% CI, 10.3–11.5). OS decreased significantly with increasing age, with highest OS observed in the ≤ 50 age group at 18.96 months (16.92–20.86) and the lowest in the ≥ 70 age group at 4.70 months (4.07–5.29) (log rank test, p < 0.0001). Patients treated at an Academic/Research Program had the highest OS amongst the different facilities at 13.63 months (12.19–15.00) (log rank test, p < 0.0001). OS decreased significantly with increasing CDS, with the highest OS in the group with a score of 0 at 12.42 months (11.76–13.17) and the lowest in the group with a score of ≥ 3 at 2.86 months (2.17–3.78) (log rank test, p < 0.0001). Across the four BC subgroups, the HR( +)/HER2( +) group had the highest OS at 22.05 months (18.73–24.67) compared to the lowest in the HR(−)/HER2(−) at 5.62 months (5.19–6.18) (log rank test, p < 0.0001). The HR( +)/HER2(–) and HR(–)/HER2( +) subgroups had similar OS at 15.80 months (14.46–17.15) and 14.59 months (11.79–16.95), respectively. There was a trend of worsening survival with increasing number of EMS, with the 1 EMS group having the highest OS at 13.17 months (12.02–14.36) compared to the group of with ≥ 3 EMS with lowest OS at 7.59 months (6.70–8.84) (log rank test, p < 0.0001). Based on the location of the EMS, bone metastasis conferred the highest OS amongst all combinations at 16.53 months (14.82–18.40) with the combined Liver and Lung group having the lowest OS at 5.22 months (3.09–6.34) (log rank test, p < 0.0001). Across the different local BM treatment modalities, patients without any treatment had the lowest OS at 9.26 months (8.31–10.02) compared to Surgery + WBRT group which had the highest OS at 32.33 months (23.98–40.44) (log rank test, p < 0.0001). Across the different BC treatment modalities, patients without any treatment had the lowest OS at 2.1 months (1.97–2.23) compared to the Chemotherapy + Hormonal Therapy + Immunotherapy group which had the highest OS at 42.35 months (35.48–54.14) (log rank test, p < 0.0001). Last, across the treatment combinations, the lowest OS was observed in the subgroup without any treatment at 1.77 months (1.64–1.97) followed by local treatment for BM only at 2.63 months (2.33–2.96). The subgroups that received BC treatment only and combination treatment for both brain and breast entities had similar OS at 16.92 months (16.00–18.27) and 16.30 months (15.11–17.38), respectively (log rank test, p < 0.0001). Table 2 summarizes the OS across the different variables, and Figs. 1–2 show the Kaplan–Meier curves with risk tables.

**Table 2:** Median overall survival (OS) across age, facility type, Charlson-Deyo score, breast cancer subtype, number of extracranial metastatic sites, location of extracranial metastatic sites, brain metastasis treatment modality, breast cancer treatment modality, and treatment combinations

Age	# of Cases	Median OS	95% CI
≤ 50 years	1022/1501	18.96	16.92, 20.86
51–60 years	1538/2054	12.98	11.89, 14.13
61–70 years	1651/2183	10.55	9.50, 11.83
≥ 70 years	1266/1523	4.70	4.07, 5.29
Total	5477/7261		
Log-rank test p-value	< .0001		
Facility type			
Academic/research program	1755/2445	13.63	12.19, 15.00
Community cancer program	472/611	9.89	7.75, 11.37
Comprehensive community cancer program	2097/2650	9.26	8.38, 9.89
Integrated network cancer program	864/1117	9.26	8.02, 10.55
Total	5188/6823		
Log-rank test p-value	< .0001		
Charlson Deyo score			
0	4251/5775	12.42	11.76, 13.17
1	821/1014	8.08	6.77, 9.56
2	249/291	4.90	3.38, 7.06
≥ 3	156/181	2.86	2.17, 3.78
Total	5477/7261		
Log-rank test p-value	< .0001		
Breast cancer subtype			
HR( − )/HER2( − )	1194/1367	5.62	5.19, 6.18
HR( − )/HER2( +)	547/767	14.59	11.79, 16.95
HR( +)/HER2( − )	2132/2936	15.80	14.46, 17.15
HR( +)/HER2( +)	655/998	22.05	18.73, 24.67
Total	4528/6068		
Log-rank test p-value	< .0001		
Number of extracranial metastatic sites			
Only brain	868/1200	12.00	10.38, 13.83
Brain + 1 metastatic site	1798/2374	13.17	12.02, 14.36
Brain + 2 metastatic sites	1562/2053	10.48	9.53, 11.83
Brain + ≥ 3 metastatic sites	1232/1613	7.59	6.70, 8.84
Total	5460/7240		
Log-rank test p-value	< .0001		
Location of extracranial metastatic sites			
Only brain	868/1200	12.00	10.38, 13.83
Bone	1127/1529	16.53	14.82, 18.40
Liver	133/161	6.37	4.57, 13.80
Lung	470/586	8.38	7.29, 9.63
Bone + liver	467/595	10.12	7.79, 12.09
Bone + lung	712/931	11.99	10.28, 13.73
Liver + lung	200/232	5.22	3.09, 6.34
Bone + liver + lung	671/805	6.83	5.49, 8.05
Other	812/1201	9.56	8.08, 10.91
Total	5460/7240		
Log-rank test p-value	< .0001		
Brain metastasis treatment modality			
No treatment	2816/3729	9.26	8.31, 10.02
WBRT	1725/2125	10.25	9.56, 11.10
SRS	442/651	15.41	13.24, 18.53
Surgery	246/358	19.81	14.78, 25.43
Surgery + SRS	165/249	20.5	16.26, 23.98
Surgery + WBRT	83/149	32.33	23.98, 40.44
Total	5477/7261		
Log-rank test p-value	< .0001		
Breast cancer treatment modality			
No treatment	1678/1955	2.10	1.97, 2.23
Immunotherapy	60/74	5.00	3.32, 8.41
Chemotherapy	1507/1817	10.50	9.86, 11.24
Hormonal therapy	967/1225	13.54	12.02, 15.11
Immunotherapy + hormonal therapy	103/154	23.69	17.74, 27.56
Chemotherapy + hormonal therapy	680/1071	26.38	24.71, 28.55
Chemotherapy + immunotherapy	345/647	27.56	24.77, 33.31
Chemotherapy + hormonal therapy + immunotherapy	133/307	42.35	35.48, 54.14
Total	5473/7250		
Log-rank test p-value	< .0001		
Treatment combination			
No treatment for both	1082/1275	1.77	1.64, 1.97
Treatment for brain metastasis only	600/691	2.63	2.33, 2.96
Treatment for breast cancer only	1734/2454	16.92	16.00, 18.27
Treatment for both	2061/2841	16.30	15.11, 17.38
Total	5477/7261		
Log-rank test p-value	< .0001		

### Median OS by treatment modality across BC subtypes

Based on BM treatment modality, the Surgery + WBRT groups achieved the highest OS across three BC subgroups at 33.35 (24.48–40.87), 48.85 (10.41–), and 15.8 (6.31–21.98), in the HR( +)/HER2( − ), HR( − )/HER2( +), and HR( − )/HER2( − ) subgroups, respectively. For the HR( +)/HER2( +) subgroup, computing the Surgery + WBRT value was not possible, and Surgery + SRS achieve the highest OS at 42.25 (12.98–) (log rank test, p < 0.0001). Based on BC treatment modality, the Chemotherapy + Hormonal therapy + Immunotherapy groups achieved the highest OS across three BC subgroups at 55.13 (35.58–), 42.35 (36.04–55.36), and 31.34 (7.82–), for the HR( +)/HER2( − ), HR( +)/HER2( +), and HR( − )/HER2( +) subgroups, respectively. For the HR( − )/HER2( − ) subgroup, Chemotherapy + Immunotherapy achieved the highest OS at 11.7 (9.46–16.72) (log rank test, p < 0.0001). Based on treatment combinations, receiving local and systemic treatment combined for both BM and BC achieved the highest OS at 19.02 (17.08–20.70), 28.94 (24.77–35.29), 19.42 (16.95–23.36), and 8.84 (7.85–9.79) for the HR( +)/HER2( − ), HR( +)/HER2( +), HR( − )/HER2( +), and HR( − )/HER2( − ) subgroups, respectively (log rank test, p < 0.0001). Table 3 summarizes the OS across the four BC subgroups, and supplementary Figs. 3–6 show the Kaplan–Meier curves with risk tables.

**Table 3:** Median overall survival (OS) for the four breast cancer subgroups across the different treatment modalities

BC subtype	HR( +)/HER2( − )			HR( +)/HER2( +)			HR( − )/HER2( +)			HR( − )/HER2( − )		
BM treatment modality	# of Cases	OS	95% CI	# of Cases	OS	95% CI	# of Cases	OS	95% CI	# of Cases	OS	95% CI
No treatment	1155/1615	14.88	13.17, 17.02	323/477	17.74	14.09, 22.34	237/329	11.56	7.85, 16.20	528/597	4.5	3.81, 5.03
WBRT	613/767	13.08	10.87, 14.39	240/331	18.83	14.23, 22.77	208/278	15.74	12.58, 18.43	452/498	6.08	5.36, 6.87
SRS	174/257	18.53	13.86, 22.77	48/97	39.79	28.75,	51/84	16.82	10.45, 30.65	107/134	8.5	5.49, 12.35
Surgery	109/159	25.43	19.35, 30.72	21/39	35.98	14.36, 53.26	25/34	10.02	4.80, 19.55	41/51	6.7	4.27, 11.37
Surgery + SRS	52/87	28.12	19.84, 44.09	17/33	42.25	12.98,	18/25	24.08	5.30, 59.17	43/53	7.46	4.44, 16.16
Surgery + WBRT	29/51	33.35	24.48, 40.87	6/21		29.11,	8/17	48.85	10.41,	23/34	15.8	6.31, 21.98
Total	2132/2936			655/998			547/767			1194/1367		
Log-rank test p-value	< .0001			< .0001			0.0209			< 0.0001		
BC treatment modality												
No treatment	463/565	2.69	2.43, 3.32	143/174	2.56	1.94, 3.02	130/152	1.77	1.45, 2.20	441/481	2.07	1.91, 2.30
Immunotherapy (I)	4/6	18.34	1.87,	19/21	4.17	2.14, 14.75	26/32	3.94	2.90, 6.05	3/6		1.45,
Chemotherapy (C)	340/433	12.48	10.87, 14.88	110/137	18.83	11.53,23.49	205/254	15.51	12.16, 17.77	683/792	8.51	7.66, 9.23
Hormonal therapy (H)	746/957	15.38	13.54,18.20	63/70	5.6	3.22, 9.03	5/6	0.81	0.39,	11/12	3.33	0.90, 16.46
I + H	46/73	31.54	24.51, 35.38	44/65	18	10.15, 23.36	3/4	7.36	1.35,		–	–
C + H	481/785	26.91	25.36, 29.14	92/134	31.57	20.96, 40.31	13/13	14.16	6.01, 24.90	17/22	10.68	6.77, 27.24
C + I	20/31	14.42	8.90, 27.07	104/209	36.37	27.50, 51.52	156/291	30.65	22.97, 35.45	39/53	11.7	9.46, 16.72
C + H + I	31/83	55.13	35.58,	77/184	42.35	36.04, 55.36	14-Sep	31.34	7.82,		–	–
Total	2131/2933			652/994			547/766			1194/1366		
Log-rank test p-value	< .0001			< .0001			< .0001			< .0001		
Treatment combination												
Neither BM nor BC	321/398	2.56	2.10, 3.32	85/106	2.27	1.74, 2.70	77/90	1.51	1.15, 2.0	232/248	1.87	1.58, 2.07
Only BM	143/170	3.25	2.50, 4.57	61/72	3.15	1.87, 5.26	53/63	2.07	1.64, 3.48	209/234	2.3	2.07, 2.63
Only BC	834/1217	22.24	18.96, 24.77	238/371	24.67	21.19, 28.68	160/239	19.02	14.88, 23.16	296/349	8.57	7.33, 9.89
BM + BC	834/1151	19.02	17.08, 20.70	271/449	28.94	24.77, 35.29	257/375	19.42	16.95, 23.36	457/536	8.84	7.85, 9.79
Total	2132/2936			655/998			547/767			1194/1367		
Log-rank test p-value	< .0001			< .0001			< .0001			< .0001		

### Cox regression model

Univariate analyses were performed on 14 explanatory variables, and significant variables were computed to a multivariate cox regression model to find hazard ratios [HR (95% CI), p–value]. On multivariate analysis, older age was associated with increased risk of death. Compared to ≤ 50–year age–group, the 51–60 year and ≥ 70–year age groups had higher risk of death [1.17(1.04–1.31), p = 0.0099)] and [1.53(1.31–1.79), p < 0.0001], respectively. Patients with races other than White had lower risk of death compared to White patients [0.78(0.63–0.96), p = 0.0216]. Hispanic patients had lower risk of death compared to non–Hispanic patients [0.72(0.60–0.86), p = 0.0003]. Compared to patients with private insurance, those who were un–insured [1.38(1.18–1.61), p < 0.0001], on Medicaid [1.28(1.14–1.43), p < 0.0001], and on Medicare [1.20(1.07–1.34), p = 0.0013] had higher risks of death. Patients with a median income of < $40,227 had higher risk of death compared to ˃$63,333 [1.22(1.06–1.40), p = 0.0058], while high school degree was not significantly associated with survival. Compared to academic/research program facilities, CCCP [1.15(1.05–1.26), p = 0.0018], and integrated network cancer programs [1.21(1.08–1.36), p = 0.0012] had higher risks of death. Compared to patients with no comorbidities, higher CDS correlated with higher risks of death at [1.13(1.02–1.26), p = 0.0249], [1.32(1.09–1.60), p = 0.0041], and [1.74(1.39–2.18), p < 0.0001] for the 1, 2, and ≥ 3 score groups, respectively. Compared to patients diagnosed in 2018–2020, those diagnosed earlier in 2010–2011 [1.25(1.08–1.45), p = 0.0029] and 2014–2015 [1.20(1.03–1.39), p = 0.0164] had higher risk of death. The three BC subgroups had lower risk of death compared to the triple negative group, with the HR( +)/HER2( +) group having the best outcome with the lowest risk [0.43(0.38–0.49), p < 0.0001]. The location and number of EMS was significantly correlated with survival. Compared to only brain, bone + liver + lung [2.06(1.78–2.38), p < 0.0001] had the highest risk of death, followed by liver + lung [1.97(1.59–2.44), p < 0.0001], bone + liver [1.96(1.67–2.31), p < 0.0001], liver [1.88(1.45–2.45), p < 0.0001], other combinations [1.85(1.58–2.18), p < 0.0001], bone + lung [1.41(1.21–1.63), p < 0.0001], lung [1.31(1.12–1.53), p = 0.0009], and bone [1.31(1.15–1.49), p < 0.0001]. Compared to patients who received treatment for both breast and brain entities, patients who had no treatment for either [2.65(2.36–2.98), p < 0.0001] and treatment for BM only [2.30(2.00–2.63), p < 0.0001] were significantly more likely to die. Treatment for BC only was not statistically significant (p = 0.0920). Table 4 summarizes the results of the univariate and multivariate cox regression models^a^.

**Table 4:** Univariate and multivariate cox regression models for variables predicting risk of death in the patient cohort

Cox regression model	Univariate		Multivariate	
Variable	HR (95% CI)	p-value	HR (95% CI)	p-value
Age				
≤ 50 years (ref)	1	–	1	–
51–60	1.25(1.16–1.36)	< 0.0001	1.17(1.04–1.31)	0.0099
61–70	1.36(1.26–1.47)	< 0.0001	1.11(0.98–1.27)	0.0935
≥ 70	2.05(1.88–2.22)	< 0.0001	1.53(1.31–1.79)	< 0.0001
Sex				
Female (ref.)	1	–		
Male	1.15(0.90–1.46)	0.2751		
Race				
White (ref.)	1	–	1	–
Black	1.09(1.02–1.16)	0.0167	1.04(0.94–1.15)	0.4849
Other	0.76(0.66–0.88)	0.0002	0.78(0.63–0.96)	0.0216
Ethnicity				
Non-Hispanic (ref.)	1	–	1	–
Hispanic	0.65(0.58–0.73)	< 0.0001	0.72(0.60–0.86)	0.0003
Insurance status				
Private insurance/managed care (ref.)	1	–	1	–
Not insured	1.37(1.23–1.52)	< 0.0001	1.38(1.18–1.61)	< 0.0001
Medicaid	1.17(1.08–1.27)	0.0002	1.28(1.14–1.43)	< 0.0001
Medicare	1.57(1.48–1.68)	< 0.0001	1.20(1.07–1.34)	0.0013
Median income quartiles (2012–2016)				
> $63,333 (ref.)	1	–	1	–
$50,354–$63,332	1.12(1.04–1.21)	0.0022	1.10(0.98–1.23)	0.1031
$40,227–$50,353	1.16(1.07–1.25)	0.0002	1.11(0.98–1.26)	0.0924
< $40,227	1.17(1.08–1.26)	< 0.0001	1.22(1.06–1.40)	0.0058
Percent no high school degree quartiles (2012–2016)				
< 6.3% (ref.)	1	–	1	–
6.3%–10.8%	1.08(1.00–1.17)	0.053	1.02(0.90–1.14)	0.7999
10.9%–17.5%	1.16(1.07–1.26)	0.0004	1.05(0.92–1.20)	0.4946
> 17.6%	1.05(0.97–1.14)	0.2211	0.90(0.77–1.05)	0.1804
Facility type				
Academic/research program (ref.)	1	–	1	–
Community cancer program	1.21(1.09–1.34)	0.0002	1.06(0.92–1.22)	0.3907
Comprehensive community cancer program	1.24(1.16–1.32)	< 0.0001	1.15(1.05–1.26)	0.0018
Integrated network cancer program	1.26(1.16–1.37)	< 0.0001	1.21(1.08–1.36)	0.0012
Charlson Deyo score				
0 (ref.)	1	–	1	–
1	1.26(1.17–1.36)	< 0.0001	1.13(1.02–1.26)	0.0249
2	1.65(1.45–1.88)	< 0.0001	1.32(1.09–1.60)	0.0041
≥ 3	1.92(1.63–2.25)	< 0.0001	1.74(1.39–2.18)	< 0.0001
Year of diagnosis				
2010	1.32(1.18–1.49)	< 0.0001		
2011	1.23(1.10–1.38)	0.0004		
2012	1.16(1.04–1.31)	0.0108		
2013	1.15(1.03–1.30)	0.0156		
2014	1.19(1.06–1.33)	0.0033		
2015	1.10(0.98–1.24)	0.0965		
2016	1.10(0.98–1.24)	0.1067		
2017	1.00(1.00–1.00)			
2018	1.13(1.01–1.27)	0.0385		
2019	1.00(1.00–1.00)			
2020 (ref.)	1	–		
Year of diagnosis (regrouped)				
2010–2011	1.20(1.10–1.30)	< 0.0001	1.25(1.08–1.45)	0.0029
2012–2013	1.09(1.00–1.18)	0.044	1.05(0.91–1.22)	0.4896
2014–2015	1.08(0.99–1.16)	0.0774	1.20(1.03–1.39)	0.0164
2016–2017	1.04(0.94–1.15)	0.5058	1.02(0.87–1.19)	0.8482
2018–2020 (ref.)	1	–	1	–
Histology				
Ductal (ref.)	1	–		
Lobular	1.03(0.92–1.14)	0.6592		
Other	1.20(1.13–1.27)	< 0.0001		
Grade				
1 (ref.)	1	–	1	
2	1.15(1.03–1.28)	0.0124	1.04(0.91–1.19)	0.547
3	1.40(1.26–1.56)	< 0.0001	1.16(1.00–1.35)	0.0551
Tumor size				
> 3 cm(ref.)	1	–		
2–3 cm	1.02(0.93–1.11)	0.73		
1–2 cm	1.02(0.93–1.12)	0.6889		
< 1 cm	0.99(0.89–1.11)	0.8897		
Lympho-vascular invasion				
0 (ref.)	1	–		
1	1.01(0.92–1.11)	0.8672		
Breast cancer subtype				
HR( − )/HER2( − ) (ref.)	1	–	1	-
HR( − )/HER2( +)	0.52(0.47–0.58)	< 0.0001	0.58(0.51–0.66)	< 0.0001
HR( +)/HER2( − )	0.51(0.48–0.55)	< 0.0001	0.54(0.49–0.60)	< 0.0001
HR( +)/HER2( +)	0.41(0.37–0.45)	< 0.0001	0.43(0.38–0.49)	< 0.0001
Location of extracranial metastatic sites				
Only brain (ref.)	1	–	1	–
Bone	0.96(0.88–1.05)	0.3799	1.31(1.15–1.49)	< 0.0001
Bone + liver	1.22(1.09–1.37)	0.0005	1.96(1.67–2.31)	< 0.0001
Bone + liver + lung	1.44(1.30–1.60)	< 0.0001	2.06(1.78–2.38)	< 0.0001
Bone + lung	1.07(0.97–1.19)	0.1576	1.41(1.21–1.63)	< 0.0001
Liver	1.27(1.06–1.52)	0.0107	1.88(1.45–2.45)	< 0.0001
Liver + lung	1.73(1.48–2.02)	< 0.0001	1.97(1.59–2.44)	< 0.0001
Lung	1.34(1.20–1.50)	< 0.0001	1.31(1.12–1.53)	0.0009
Other	1.22(1.11–1.35)	< 0.0001	1.85(1.58–2.18)	< 0.0001
Treatment combination				
Treatment for both (ref.)	1	–	1	–
Treatment for breast cancer only	0.99(0.93–1.06)	0.7528	0.93(0.85–1.01)	0.092
Treatment for brain metastasis only	2.42(2.21–2.65)	< 0.0001	2.30(2.00–2.63)	< 0.0001
No treatment for both	3.14(2.91–3.38)	< 0.0001	2.65(2.36–2.98)	< 0.0001

The model included fourteen explanatory variables (age, race, ethnicity, insurance status, median household income quartile 2012–2016, percent of no high school degree, Charlson Deyo Score, histology, grade, breast cancer subtype, metastasis location sites, treatment combinations, and year of diagnosis)

^a^Univariate logistic regressions ran first. Sex, tumor size, and lympho-vascular invasion all not significant so not included in multivariate model. Histology (p = 0.1024) was eliminated by backward elimination. Model set at 0.1 cutoff

## Discussion

In this analysis, we identified several factors contributing to prognosis of patients presenting with de novo BCBM including age, facility type, CDS, BC subtype, number and location of EMS, and local and systemic treatment modalities. Younger age, treatment at an academic/research program, lower CDS, triple positive BC status, having only one EMS, receiving surgery and WBRT, receiving Chemotherapy + Hormonal Therapy + Immunotherapy, and receiving combined BM and BC therapies were all associated with improved OS.

This data is consistent with another retrospective analysis including n = 1366 patients with de novo BCBM patients from the Surveillance, Epidemiology, and End Results (SEER) database between 2015 and 2019, by Yaning et al. finding median OS to be 12.0 months (10.4–13.6), which is very similar to our cohort’s value of 10.9 months [23]. Furthermore, the authors identified similar trends in subgroup survival, with the HR( +)/HER2( +) group having the best OS at 19.0 months (11.8–26.2) and the HR( − )/HER2( − ) having the worst OS at 7.0 months (5.4–8.6), both of which overlap with our results. Moreover, there was a similar trend in the OS of patients based on the metastatic sites with the bone only group having the longest OS (17.0 vs 16.5 months in our cohort) and all three sites (bone + liver + lung) having the lowest OS at 8.0 months (5.4–10.6) compared to 6.8 months in our cohort. Lastly, the OS decreased with increasing number of EMS like what was observed in our cohort. Similar trends were also observed in another study conducted on 248 patients with de novo BCBM between 2010 and 2018 from the SEER database [24]. In our analysis, OS decreased with increasing age, number of comorbidities, and number of EMS, which is in line with previously noted studies.

Overall, Surgery + WBRT yielded the best survival benefit amongst BM treatments, and these findings were also consolidated in the BC subgroup analysis. This is in line with the findings of the GPA study by Sperduto et al. which found that Surgery + WBRT treatment achieved the highest OS amongst all other combinations in BCBM patients at 25 months [2]. On the other hand, a recent systematic review on radiation therapy for BM identified five randomized trials conducted on post–surgical radiotherapy (SRS or WBRT) and found no differences in OS in the pooled results [25]. A growing number of clinical trials are ongoing to explore the best treatment modality for the local treatment of BCBM patients [6].

Overall, Chemotherapy + Hormonal Therapy + Immunotherapy yielded the best survival benefit amongst all BC treatments, findings also observed in the BC subgroup analysis. Of note, immunotherapy consistently improved survival across all the BC subtypes when added to systemic therapy. For example, in the HR( +)/HER2( − ) subgroup, adding targeted therapy more than doubled survival when added to the hormonal therapy alone group (from 15.38 to 31.54 months) and to the Chemotherapy + Hormonal therapy group (from 26.91 to 55.13 months). There is a growing number of studies and clinical trials that are investigating promising targeted and biologic therapies to target BCBM and shown survival benefits [12] which could explain the improved survival outcomes in our analysis with the addition of anti–HER2 therapy and other targeted therapies. Some of the drugs being explored include the anti HER2 targeting antibodies including: Trastuzumab [26, 27], Trastuzumab Emtansine [28, 29], Trastuzumab Deruxtecan [30], and Pertuzumab [31]; tyrosine kinase inhibitors including: Lapatinib [32–35], Neratinib [36–38], Afatinib [39], Tucatinib [40], Taselisib [41], Alpelisib [42], Buparlisib [43]; and CDK 4/6 inhibitors including: Palbociclib [44], Ribociclib [45], and Abemaciclib [46]; among other classes of targeted therapies. Unfortunately, the biologic agents used in treatment of the BCBM patient cohort are not available in the NCDB, but the trend of improved survival speaks to the rapid development of new targeted therapies that are currently under study. One example is the approval of Pembrolizumab for neoadjuvant and adjuvant treatment of patients with high–risk early–stage triple–negative BC in 2021 [47]. The study at hand is limited to 2020 and hence outcomes may improve even more for triple negative breast cancer in the coming years with more targeted therapies approved.

In the combined treatment analysis, receiving treatment for BM alone did not seem to prolong survival. Furthermore, treating BC alone achieved similar survival to treating both BC and BM. This suggests that the major therapeutic contributor to OS in de novo BCBM patients is the treatment of the underlying primary tumor rather than the BM itself. This finding is further supported by the findings of the multivariable cox regression model which integrates all the variables to identify and validate the individual survival benefits. In the model, treatment of BM alone increased the risk of death 2.3 folds compared to receiving dual treatment, which suggests that it is the BC treatment that confers any survival benefit.

## Limitations

The study at hand has several limitations by virtue of it being conducted on a retrospective database which impedes control of certain variables. Furthermore, the NCDB does not provide information about relevant prognostic indicators identified in many studies such as number and size of BM, KPS, and the type of chemotherapy and targeted therapy received. Additionally, it was not possible to delineate the extent of BM surgery, and the radiation dose and number of treatment fractions to the BM in the analysis. Last, the NCDB provides information only about de novo BM and not recurrent BM. Recurrent BM constitutes a bigger percentage of BM and remains an important factor to consider when predicting prognosis. Despite these limitations, this remains, to the best of our knowledge, the biggest cohort of de novo BCBM patients to date and provides valuable information for clinical practice.

## Conclusion

We retrospectively analyzed the biggest cohort of de novo BCBM patients exploring clinical and therapeutic factors associated with survival. Our results maintain the short survival of BCBM patients while also providing subgroup specific values that can guide clinical decision making. The BM–specific treatment that yielded the best survival outcomes was surgery combined with WBRT, and targeted therapy improved survival when added to systemic therapy across all subgroups. Further analysis showed that treating BM alone may decrease survival compared to receiving treatment for both BM and BC indicating that the primary disease is the main predictor of survival, and the BM management may serve a palliative role. Prospective studies are needed to consolidate these findings and to further highlight the role of targeted personalized therapy in improving survival of patients with BCBM.

### Supplementary Information

Below is the link to the electronic supplementary material.

Supplementary file1 (DOCX 4035 KB)

## Data Availability

No datasets were generated or analysed during the current study.

## References

[1] Łukasiewicz S, Czeczelewski M, Forma A, Baj J, Sitarz R, Stanisławek A (2021). Breast cancer-epidemiology, risk factors, classification, prognostic markers, and current treatment strategies-an updated review. Cancers (Basel).

[2] Sperduto PW, Mesko S, Li J, Cagney D, Aizer A, Lin NU, Nesbit E, Kruser TJ, Chan J, Braunstein S (2020). Survival in patients with brain metastases: summary report on the updated diagnosis-specific graded prognostic assessment and definition of the eligibility quotient. J Clin Oncol.

[3] Daily K, Douglas E, Romitti PA, Thomas A (2021). Epidemiology of de novo metastatic breast cancer. Clin Breast Cancer.

[4] Mariotto AB, Etzioni R, Hurlbert M, Penberthy L, Mayer M (2017). Estimation of the number of women living with metastatic breast cancer in the United States. Cancer Epidemiol Biomarkers Prev.

[5] Conquer Cancer the ASCO Foundation (2022) Board CNE: breast cancer—metastatic: introduction.

[6] Watase C, Shiino S, Shimoi T, Noguchi E, Kaneda T, Yamamoto Y, Yonemori K, Takayama S, Suto A (2021). Breast cancer brain metastasis-overview of disease state, treatment options and future perspectives. Cancers (Basel).

[7] Kuksis M, Gao Y, Tran W, Hoey C, Kiss A, Komorowski AS, Dhaliwal AJ, Sahgal A, Das S, Chan KK (2021). The incidence of brain metastases among patients with metastatic breast cancer: a systematic review and meta-analysis. Neuro Oncol.

[8] Li R, Zhang K, Siegal GP, Wei S (2017). Clinicopathological factors associated with survival in patients with breast cancer brain metastasis. Hum Pathol.

[9] Wang R, Zhu Y, Liu X, Liao X, He J, Niu L (2019). The clinicopathological features and survival outcomes of patients with different metastatic sites in stage IV breast cancer. BMC Cancer.

[10] Sperduto PW, Kased N, Roberge D, Xu Z, Shanley R, Luo X, Sneed PK, Chao ST, Weil RJ, Suh J (2012). Summary report on the graded prognostic assessment: an accurate and facile diagnosis-specific tool to estimate survival for patients with brain metastases. J Clin Oncol.

[11] Horbinski C, Nabors LB, Portnow J, Baehring J, Bhatia A, Bloch O, Brem S, Butowski N, Cannon DM, Chao S (2023). NCCN guidelines insights: central nervous system cancers, version 2.2022: featured updates to the NCCN guidelines. J Natl Compr Canc Netw.

[12] Bailleux C, Eberst L, Bachelot T (2021). Treatment strategies for breast cancer brain metastases. Br J Cancer.

[13] Raghunath A, Desai K, Ahluwalia MS (2019). Current treatment options for breast cancer brain metastases. Curr Treat Options Oncol.

[14] Corti C, Antonarelli G, Criscitiello C, Lin NU, Carey LA, Cortés J, Poortmans P, Curigliano G (2022). Targeting brain metastases in breast cancer. Cancer Treat Rev.

[15] Fares J, Kanojia D, Rashidi A, Ulasov I, Lesniak MS (2020). Landscape of combination therapy trials in breast cancer brain metastasis. Int J Cancer.

[16] Nasrazadani A, Brufsky A (2020). Neratinib: the emergence of a new player in the management of HER2+ breast cancer brain metastasis. Future Oncol.

[17] Anwar M, Chen Q, Ouyang D, Wang S, Xie N, Ouyang Q, Fan P, Qian L, Chen G, Zhou E (2021). Pyrotinib treatment in patients with HER2-positive metastatic breast cancer and brain metastasis: exploratory final analysis of real-world. Multicenter Data Clin Cancer Res.

[18] Nader-Marta G, Martins-Branco D, Agostinetto E, Bruzzone M, Ceppi M, Danielli L, Lambertini M, Kotecki N, Awada A, de Azambuja E (2022). Efficacy of tyrosine kinase inhibitors for the treatment of patients with HER2-positive breast cancer with brain metastases: a systematic review and meta-analysis. ESMO Open.

[19] Sun H, Xu J, Dai S, Ma Y, Sun T (2023). Breast cancer brain metastasis: current evidence and future directions. Cancer Med.

[20] Mills MN, King W, Soyano A, Pina Y, Czerniecki BJ, Forsyth PA, Soliman H, Han HS, Ahmed KA (2022). Evolving management of HER2+ breast cancer brain metastases and leptomeningeal disease. J Neurooncol.

[21] Ivanova M, Porta FM, Giugliano F, Frascarelli C, Sajjadi E, Venetis K, Cursano G, Mazzarol G, Guerini-Rocco E, Curigliano G (2023). Breast cancer with brain metastasis: molecular insights and clinical management. Genes (Basel).

[22] Epaillard N, Bassil J, Pistilli B (2023). Current indications and future perspectives for antibody-drug conjugates in brain metastases of breast cancer. Cancer Treat Rev.

[23] He Y, Shao Y, Chen Q, Liu C, Zhu F, Liu H (2023). Brain metastasis in de novo stage IV breast cancer. Breast.

[24] Sun MS, Yun YY, Liu HJ, Yu ZH, Yang F, Xu L (2022). Brain metastasis in de novo breast cancer: an updated population-level study from SEER database. Asian J Surg.

[25] Garsa A, Jang JK, Baxi S, Chen C, Akinniranye O, Hall O, Larkin J, Motala A, Hempel S (2021). Radiation therapy for brain metastases: a systematic review. Pract Radiat Oncol.

[26] Park YH, Park MJ, Ji SH, Yi SY, Lim DH, Nam DH, Lee JI, Park W, Choi DH, Huh SJ (2009). Trastuzumab treatment improves brain metastasis outcomes through control and durable prolongation of systemic extracranial disease in HER2-overexpressing breast cancer patients. Br J Cancer.

[27] Pestalozzi BC, Holmes E, de Azambuja E, Metzger-Filho O, Hogge L, Scullion M, Láng I, Wardley A, Lichinitser M, Sanchez RI (2013). CNS relapses in patients with HER2-positive early breast cancer who have and have not received adjuvant trastuzumab: a retrospective substudy of the HERA trial (BIG 1–01). Lancet Oncol.

[28] Krop IE, Lin NU, Blackwell K, Guardino E, Huober J, Lu M, Miles D, Samant M, Welslau M, Diéras V (2015). Trastuzumab emtansine (T-DM1) versus lapatinib plus capecitabine in patients with HER2-positive metastatic breast cancer and central nervous system metastases: a retrospective, exploratory analysis in EMILIA. Ann Oncol.

[29] Montemurro F, Delaloge S, Barrios CH, Wuerstlein R, Anton A, Brain E, Hatschek T, Kelly CM, Peña-Murillo C, Yilmaz M (2020). Trastuzumab emtansine (T-DM1) in patients with HER2-positive metastatic breast cancer and brain metastases: exploratory final analysis of cohort 1 from KAMILLA, a single-arm phase IIIb clinical trial. Ann Oncol.

[30] Modi S, Saura C, Yamashita T, Park YH, Kim SB, Tamura K, Andre F, Iwata H, Ito Y, Tsurutani J (2020). Trastuzumab deruxtecan in previously treated HER2-positive breast cancer. N Engl J Med.

[31] Swain SM, Baselga J, Miles D, Im YH, Quah C, Lee LF, Cortés J (2014). Incidence of central nervous system metastases in patients with HER2-positive metastatic breast cancer treated with pertuzumab, trastuzumab, and docetaxel: results from the randomized phase III study CLEOPATRA. Ann Oncol.

[32] Lin NU, Diéras V, Paul D, Lossignol D, Christodoulou C, Stemmler HJ, Roché H, Liu MC, Greil R, Ciruelos E (2009). Multicenter phase II study of lapatinib in patients with brain metastases from HER2-positive breast cancer. Clin Cancer Res.

[33] Bachelot T, Romieu G, Campone M, Diéras V, Cropet C, Dalenc F, Jimenez M, Le Rhun E, Pierga JY, Gonçalves A (2013). Lapatinib plus capecitabine in patients with previously untreated brain metastases from HER2-positive metastatic breast cancer (LANDSCAPE): a single-group phase 2 study. Lancet Oncol.

[34] Geyer CE, Forster J, Lindquist D, Chan S, Romieu CG, Pienkowski T, Jagiello-Gruszfeld A, Crown J, Chan A, Kaufman B (2006). Lapatinib plus capecitabine for HER2-positive advanced breast cancer. N Engl J Med.

[35] Lin NU, Freedman RA, Ramakrishna N, Younger J, Storniolo AM, Bellon JR, Come SE, Gelman RS, Harris GJ, Henderson MA (2013). A phase I study of lapatinib with whole brain radiotherapy in patients with Human Epidermal Growth Factor Receptor 2 (HER2)-positive breast cancer brain metastases. Breast Cancer Res Treat.

[36] Awada A, Colomer R, Inoue K, Bondarenko I, Badwe RA, Demetriou G, Lee SC, Mehta AO, Kim SB, Bachelot T (2016). Neratinib plus paclitaxel vs trastuzumab plus paclitaxel in previously untreated metastatic ERBB2-positive breast cancer: the NEfERT-T randomized clinical trial. JAMA Oncol.

[37] Freedman RA, Gelman RS, Anders CK, Melisko ME, Parsons HA, Cropp AM, Silvestri K, Cotter CM, Componeschi KP, Marte JM (2019). TBCRC 022: a phase II trial of neratinib and capecitabine for patients with human epidermal growth factor receptor 2-positive breast cancer and brain metastases. J Clin Oncol.

[38] Saura C, Oliveira M, Feng YH, Dai MS, Chen SW, Hurvitz SA, Kim SB, Moy B, Delaloge S, Gradishar W (2020). Neratinib plus capecitabine versus lapatinib plus capecitabine in HER2-positive metastatic breast cancer previously treated with ≥ 2 HER2-directed regimens: phase III NALA trial. J Clin Oncol.

[39] Cortés J, Dieras V, Ro J, Barriere J, Bachelot T, Hurvitz S, Le Rhun E, Espié M, Kim SB, Schneeweiss A (2015). Afatinib alone or afatinib plus vinorelbine versus investigator's choice of treatment for HER2-positive breast cancer with progressive brain metastases after trastuzumab, lapatinib, or both (LUX-Breast 3): a randomised, open-label, multicentre, phase 2 trial. Lancet Oncol.

[40] Murthy RK, Loi S, Okines A, Paplomata E, Hamilton E, Hurvitz SA, Lin NU, Borges V, Abramson V, Anders C (2020). Tucatinib, trastuzumab, and capecitabine for HER2-positive metastatic breast cancer. N Engl J Med.

[41] Dent S, Cortés J, Im YH, Diéras V, Harbeck N, Krop IE, Wilson TR, Cui N, Schimmoller F, Hsu JY (2021). Phase III randomized study of taselisib or placebo with fulvestrant in estrogen receptor-positive, PIK3CA-mutant, HER2-negative, advanced breast cancer: the SANDPIPER trial. Ann Oncol.

[42] André F, Ciruelos E, Rubovszky G, Campone M, Loibl S, Rugo HS, Iwata H, Conte P, Mayer IA, Kaufman B (2019). Alpelisib for PIK3CA-mutated, hormone receptor-positive advanced breast cancer. N Engl J Med.

[43] Di Leo A, Johnston S, Lee KS, Ciruelos E, Lønning PE, Janni W, O'Regan R, Mouret-Reynier MA, Kalev D, Egle D (2018). Buparlisib plus fulvestrant in postmenopausal women with hormone-receptor-positive, HER2-negative, advanced breast cancer progressing on or after mTOR inhibition (BELLE-3): a randomised, double-blind, placebo-controlled, phase 3 trial. Lancet Oncol.

[44] Shah AN, Santa-Maria CA, Mukhija D, Shah N, Kang AK, Kumthekar P, Burdett K, Chandra S, Chang J, Tsarwhas D (2023). A phase II single-arm study of palbociclib in patients with HER2-positive breast cancer with brain metastases and analysis of ctDNA in patients with active brain metastases. Clin Breast Cancer.

[45] Hortobagyi GN, Stemmer SM, Burris HA, Yap YS, Sonke GS, Paluch-Shimon S, Campone M, Blackwell KL, André F, Winer EP (2016). Ribociclib as first-line therapy for HR-positive, advanced breast cancer. N Engl J Med.

[46] Tolaney SM, Sahebjam S, Le Rhun E, Bachelot T, Kabos P, Awada A, Yardley D, Chan A, Conte P, Diéras V (2020). A phase II study of abemaciclib in patients with brain metastases secondary to hormone receptor-positive breast cancer. Clin Cancer Res.

[47] Shah M, Osgood CL, Amatya AK, Fiero MH, Pierce WF, Nair A, Herz J, Robertson KJ, Mixter BD, Tang S (2022). FDA approval summary: pembrolizumab for neoadjuvant and adjuvant treatment of patients with high-risk early-stage triple-negative breast cancer. Clin Cancer Res.

