# Supplementary material for: Clinico–pathologic factors and survival of patients with breast cancer diagnosed with de novo brain metastasis: a national cancer database analysis
Source: Breast Cancer Res Treat. 2024 Apr 29;206(3):527–41. doi: 10.1007/s10549-024-07321-x (PMC11208224; doi:10.1007/s10549-024-07321-x)
Supplement: Supplementary file 1 — Supplementary file1 (DOCX 4035 KB) [file 10549_2024_7321_MOESM1_ESM.docx]

**Figure 3.** Kaplan-Meier plots of overall survival for breast cancer patients with brain metastases in the HR(+)/HER2(-) subgroup stratified by (a) brain metastases treatment modality, (b) breast cancer treatment modality, and (c) combination of both breast cancer and brain metastases treatments.


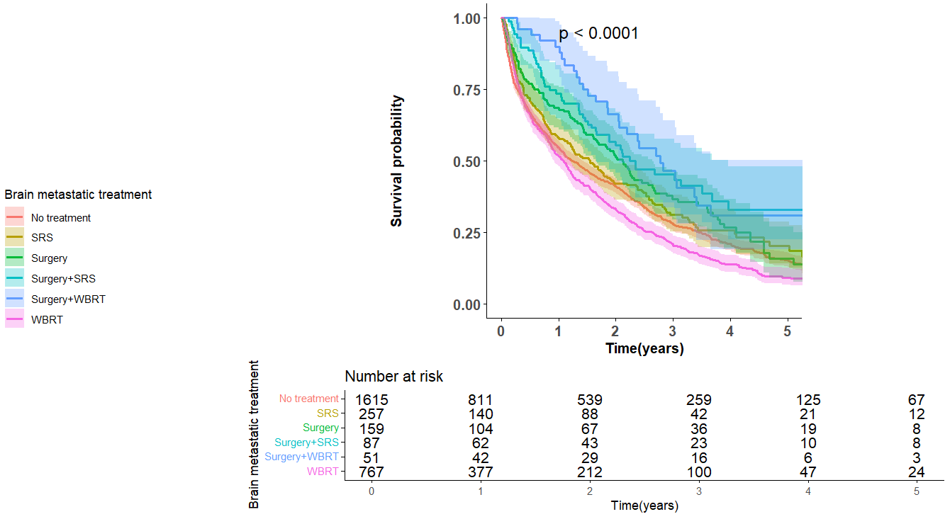

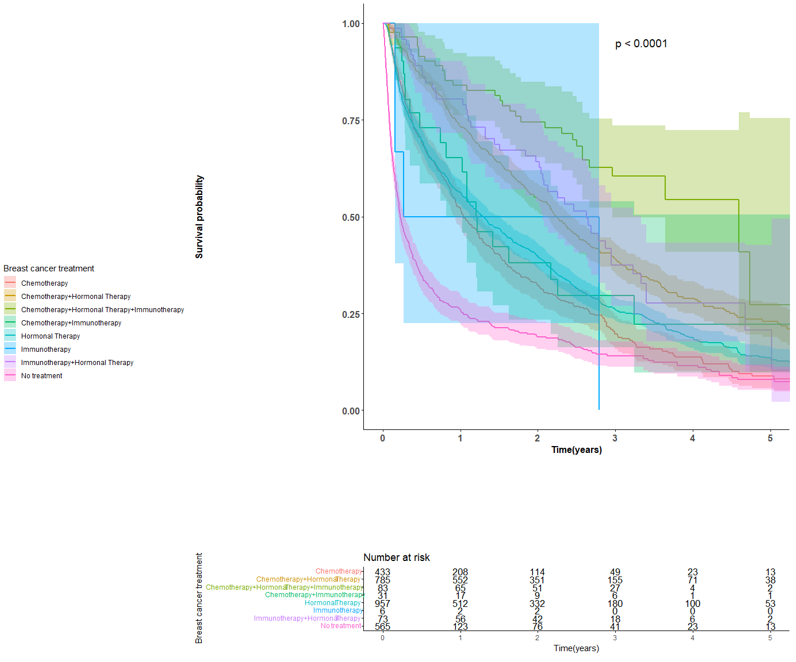

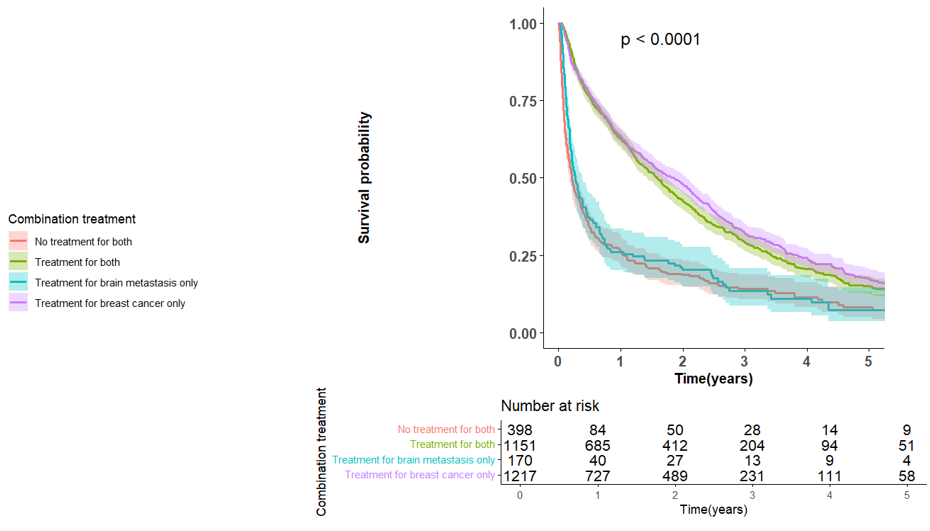


1) HR(+)/HER2(-)

(a)

(b)

(c)

**Figure 4.** Kaplan-Meier plots of overall survival for breast cancer patients with brain metastases in the HR(+)/HER2(+) subgroup stratified by (a) brain metastases treatment modality, (b) breast cancer treatment modality, and (c) combination of both breast cancer and brain metastases treatments.


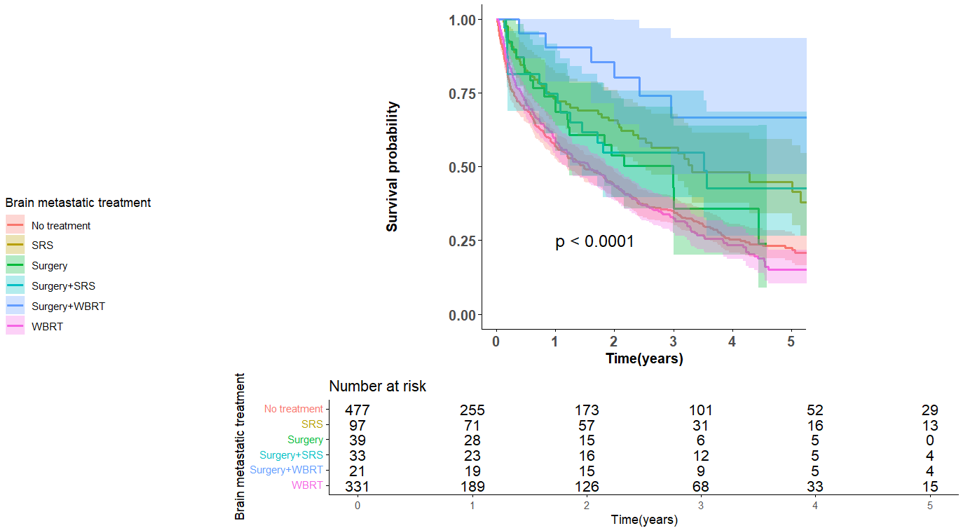

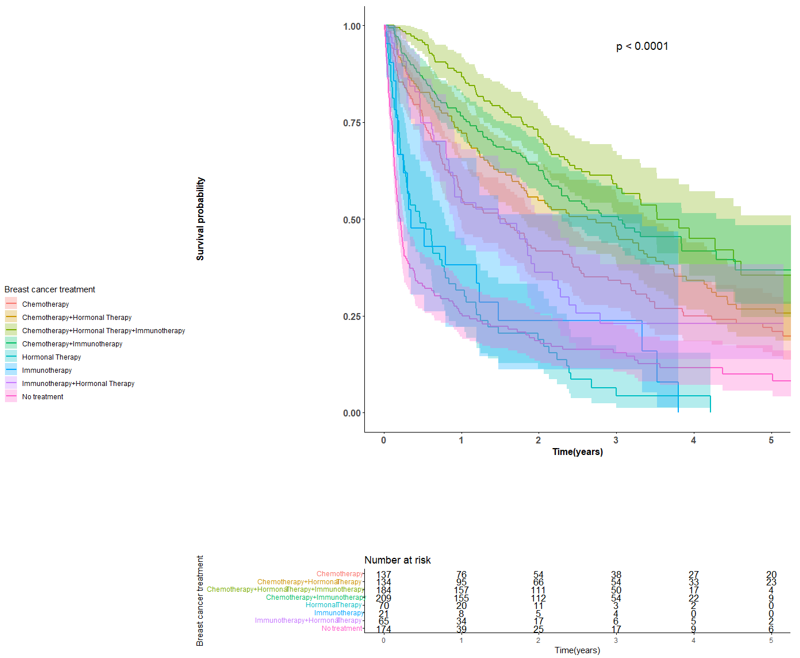

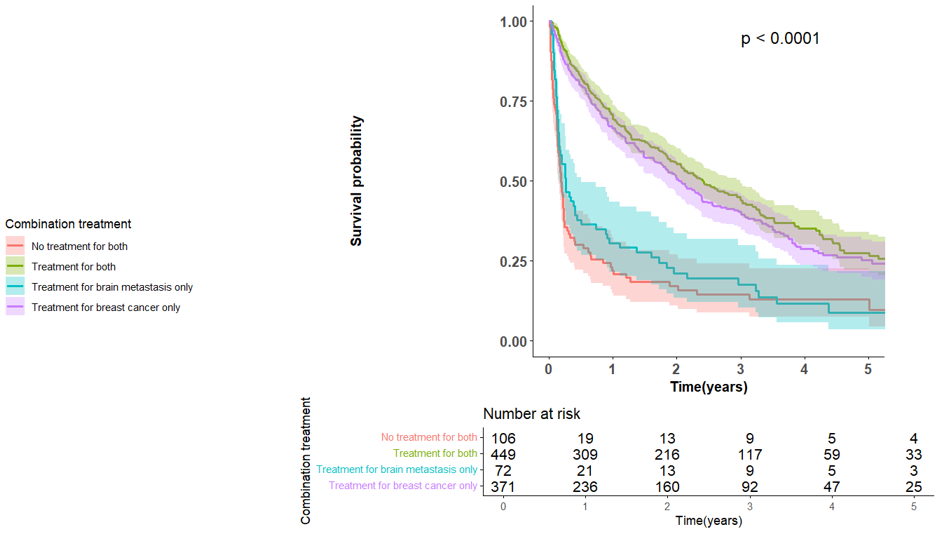


2) HR(+)/HER2(+)

(a)

(b)

(c)

**Figure 5.** Kaplan-Meier plots of overall survival for breast cancer patients with brain metastases in the HR(-)/HER2(+) subgroup stratified by (a) brain metastases treatment modality, (b) breast cancer treatment modality, and (c) combination of both breast cancer and brain metastases treatments.


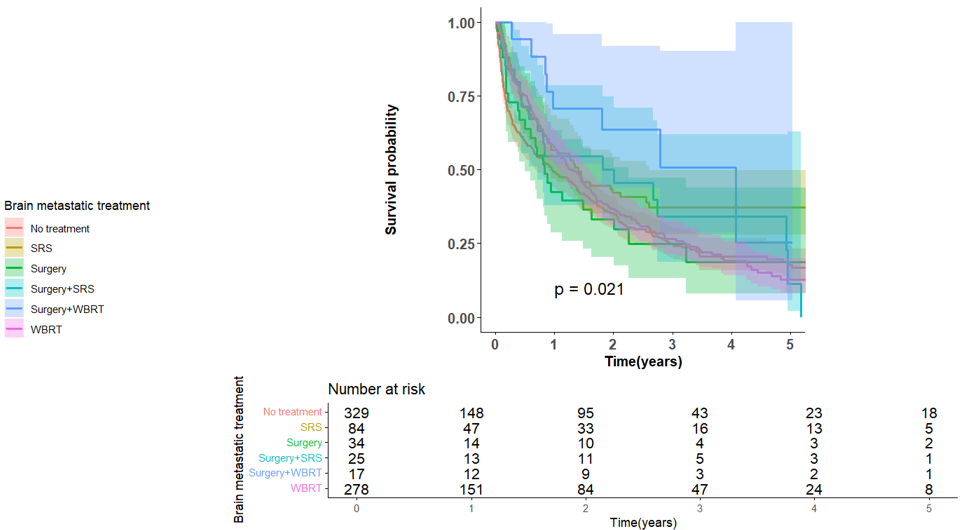

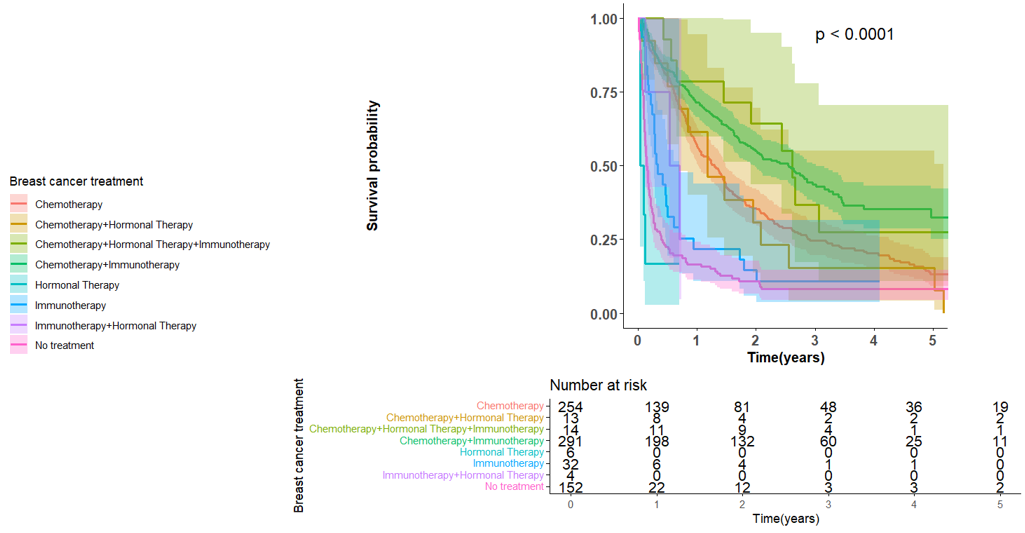

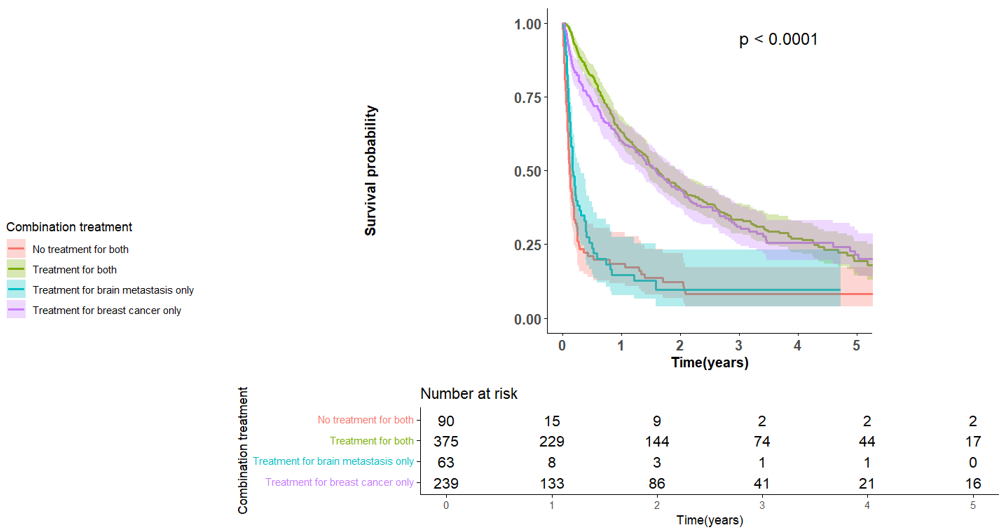


3) HR(-)/HER2(+)

(a)

(b)

(c)

**Figure 6.** Kaplan-Meier plots of overall survival for breast cancer patients with brain metastases in the HR(-)/HER2(-) subgroup stratified by (a) brain metastases treatment modality, (b) breast cancer treatment modality, and (c) combination of both breast cancer and brain metastases treatments.


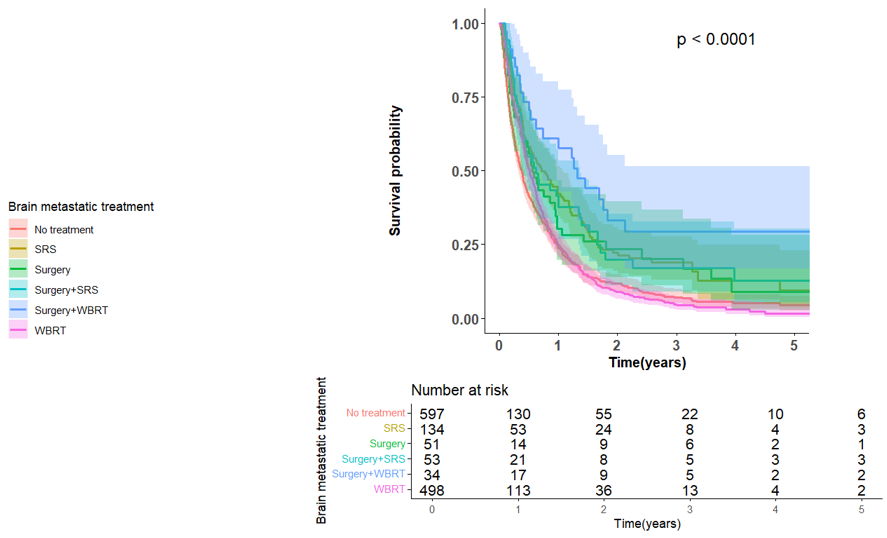

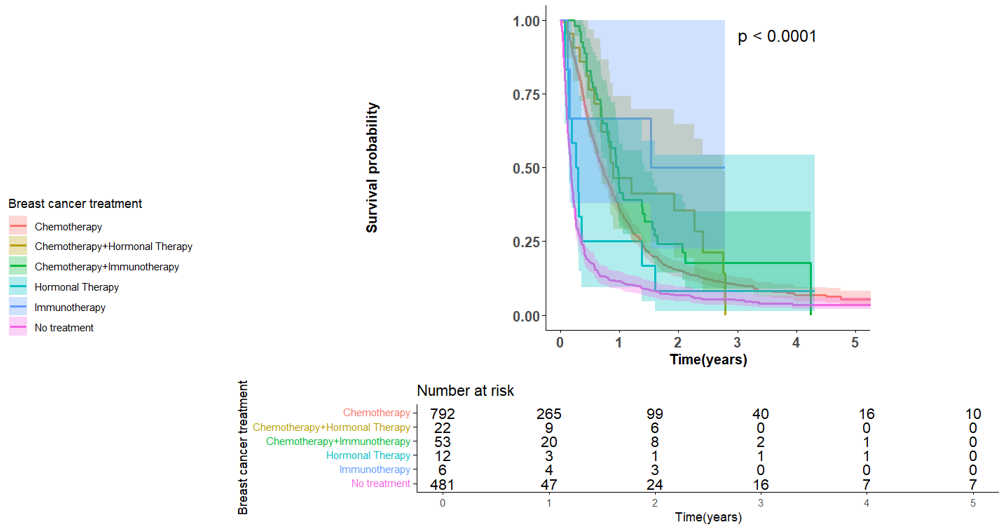

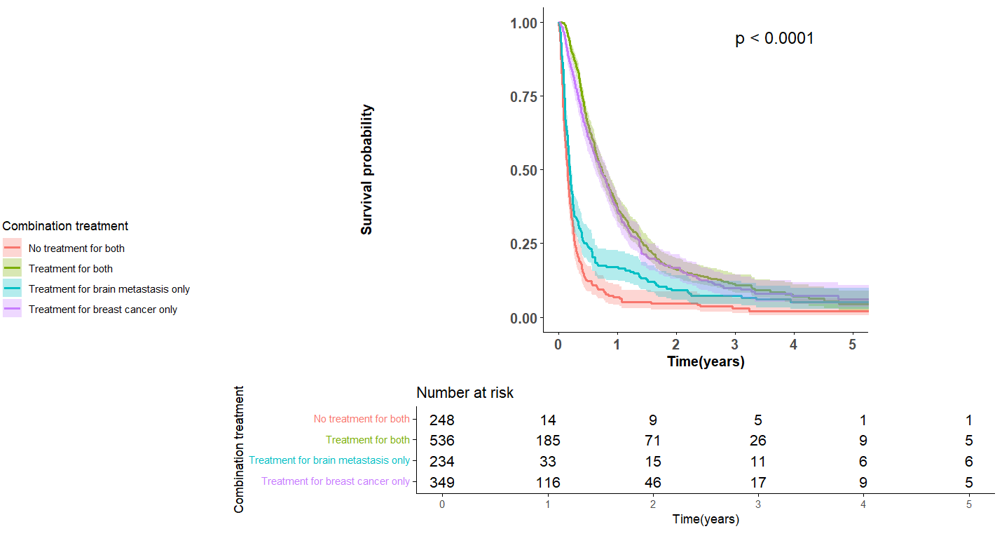


4) HR(-)/HER2(-)

(a)

(b)

(c)
